# Incremental Adjustments to Amount of Thickening Agent in Beverages: Implications for Clinical Practitioners Who Oversee Nutrition Care Involving Thickened Liquids

**DOI:** 10.3390/foods8020074

**Published:** 2019-02-14

**Authors:** Jane Mertz Garcia, Edgar Chambers

**Affiliations:** 1 Communication Sciences & Disorders, School of Family Studies & Human Services, Kansas State University, 1405 Campus Creek Road, Manhattan, KS 66506, USA; 2 Center for Sensory Analysis and Consumer Behavior, 1310 Research Park Dr., Manhattan, KS 66502, USA; eciv@ksu.edu

**Keywords:** dysphagia, swallowing, thickened beverages, liquids, viscosity, quality of care

## Abstract

This study examined the changes in viscosity in response to small alterations in the amount of a thickening agent mixed with three commonly thickened beverages. A total of 11 incremental adjustments in the amount of a starch-based thickening agent (5.0 g to 7.0 g) were made. The results showed that the incremental increases resulted in systematic changes to the liquid thickness, reflecting modifications that ranged from a nectar (mildly thick) to a honey-like (moderately thick) level of consistency. The findings emphasize the importance of the proper preparation of thickened beverages, highlighting the need for standards in training practices and the use of simple measurement tools for assuring the prescribed levels of consistency.

## 1. Introduction

The nutritional care of patients with dysphagia who consume thickened beverages requires the careful preparation of drinks to their prescribed level of modification. Basic care staff (e.g., dietary aides, cooks, and certified nursing assistants) are typically tasked with preparing modifications even though they reportedly receive little or no formal instruction about thickened liquids [1,2]. Although a number of modified beverages are currently available in ready-to-serve form, thickening liquids with commercial thickeners that require preparation by caregivers are a cornerstone of nutrition care in many settings [3,4].

One practical concern about the preparation of thickened beverages is the lack of adherence to product guidelines when determining the amount of thickening agent to mix into a beverage. Garcia et al. [2] found that many of the care staff did not look at the product label information to determine the specified amount of thickening agent. Unsurprisingly, the modifications they prepared varied considerably in terms of measured viscosity when compared to similar product/beverage combinations made in a laboratory environment using controlled conditions [2]. Even speech-language pathologists (who are experienced in dysphagia management) have difficulty preparing thickened beverages in a reliably consistent manner [5]. This inability to prepare thickened liquids consistently is a cause for concern because of the health implications linked to over- or under-thickening the beverage of patients.

Clinical practitioners are encouraged to recommend the least viscous modification that is swallowed safely, which further magnifies the importance of careful preparations [6,7,8]. Inaccuracies in preparation increase the risk of additional medical complications. For example, overly thickened beverages are more difficult for frail elders to clear from their airway, heightening the risk of pulmonary complications or aspiration pneumonia [9]. Such problems may occur in patients with impaired swallowing or a diminished ability to cough. Overly thick modifications also contribute to post-swallow residues in the pharynx [7,8]. Because the addition of a thickening agent affects the flavor and texture of a drink in a potentially negative way [10,11], caregivers who routinely overly thicken may create a modification that impacts the beverage’s acceptance. Patients with a strong dislike of the thickened beverages may, as a result, be less compliant and drink less, ultimately impacting their nutritional status. This occurs through a decreased consumption of juices, liquid nutritional supplements and other beverages. Additionally, a reduced consumption of beverages also can contribute to other medical complications such as dehydration, especially if water the consumption decreases.

Although the concern regarding preparation inaccuracies is clear, less is known about how small variations in the amount of thickening agent impact physical measures of viscosity. Park et al. [12] already evaluated various levels of gum-based thickeners, both guar and xanthan, showing that the viscosity of guar gum thickened liquids changed more rapidly than that of xanthan gum as the concentration increased, perhaps because of shear thinning. Such results suggest that in some cases inadvertent changes in thickening, such as might occur when beverages are mixed improperly, could result in large changes in viscosity. Because no work has been found examining incremental changes in starch-based thickeners, the goal of this study was to understand how incremental changes in the amount of starch-based thickening agents contributed, if at all, to viscometer readings for different modified beverages.

## 2. Materials and Methods

We selected Thick & Easy (Hormel Health Labs, Austin, MN, USA), a commercial thickening agent commonly used in modifying liquids for patients with dysphagia. The product ingredients listed modified corn starch and maltodextrin. The three beverages included water, apple juice (Musselman’s, Peach Glen, PA, USA) and cran-apple juice, a commercial blend of apple and cranberry juice (Ocean Spray, Lakeville-Middleboro, MA, USA). The beverages represented common drinks in care facilities that are easily mixed (no clumping) with a thickening agent. All have quite a low viscosity (cps 0–2). It must be noted that for this project a variety of samples could have been selected. We intentionally chose to keep the study small and focus on one thickener, a starch-based product that is widely used in dysphagia management in the United States. Gum-based thickeners are also widely used, but typically they are more difficult to mix, and this would present an added variable to this study. In addition, Park et al. [12] already evaluated various levels of gum-based thickeners.

### 2.1. Sample Preparation Procedure

**Figure 1:**
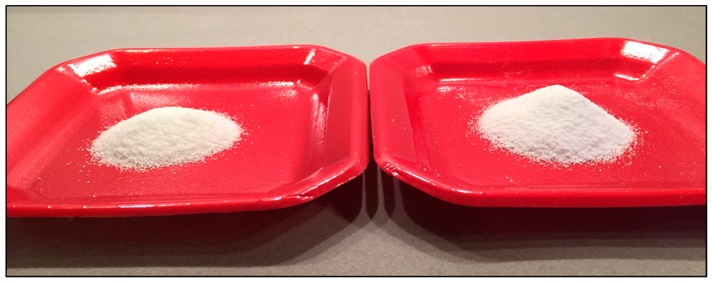
Visual comparison of 5.0 g (3 ½ tsp) and 7.0 g (5 tsp) of instant thickening agent.

The volumetric amount of liquid (120 mL) was converted into grams to ensure the same amount of beverage for each sample. The amount of thickening agent (also measured in grams) was slowly poured and stirred into the base liquid at room temperature (20–22 °C) and then continuously stirred for 40 s until it dissolved using a Barnstead Thermolyne magnetic stirring device (Cimarec 2) set to a constant speed. We systematically increased the amount of thickening agent by 0.2 g (e.g., 5.0 g, 5.2 g, 5.4 g, and 5.6 g) in each subsequent test sample. An increase of 0.2 g equated to slightly less than a 1mL (1/8 tsp) measuring spoon difference of thickening agent. Three separate samples were prepared for 11 incremental changes in thickening agent (5.0 g to 7.0 g) across beverages. The lowest to highest amount of instant thickening agent (a difference of 2.0 g) represented a difference of about 7.5 mL (by volume) of powdered thickener (equivalent to about 1½ tsp, a common measure in the US). The thickness ranged from nectar-like (mildly thick) to honey-like (moderately thick), including the border of the two thickness levels (Figure 1).

### 2.2. Viscosity Measurement

Viscosity was measured using a Brookfield RVDV- II+ viscometer using the small sample adaptor and calculated at a shear rate of 55.8 to be consistent with instrument guidelines and modification ranges described by the National Dysphagia Diet [13]. The samples were measured at room temperature (20–22 °C) after two minutes of thickening time. The measurements were collected for a total of 99 samples (11 changes in gram amount × 3 beverages × 3 replicates).

### 2.3. Statistical Analysis

We entered the data into the IBM SPSS System for Windows (IBM, New York, USA, Version 25, 2017); a “descriptive” statistics procedure was used to determine the means and standard deviations for each gram amount and for each beverage over the three replicates. The gram measurements and physical measurements of viscosity of each beverage were compared using Pearson product-moment correlation coefficients with a significance at the 0.01 level (2-tailed).

## 3. Results

**Figure 2:**
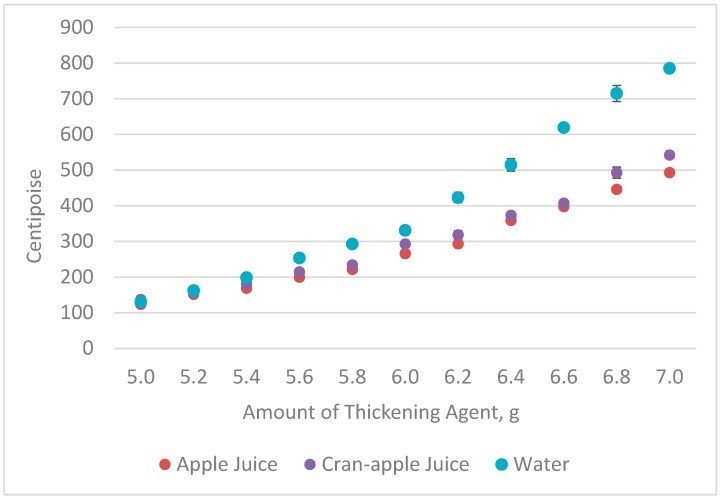
Viscosity measurements for the incremental changes in the amount of instant thickening agent (Error bars show the standard error for each mean).

Table 1 shows the average measurement of viscosity in centipoise (cP) for the three beverages prepared with 5.0 to 7.0 g of instant thickener. The mean values changed for each increase in the amount of thickening agent; higher values represented more viscous (thicker) samples. The gram measurements and physical measurements of viscosity correlated significantly for thickened water (*r* = 0.97, *p* < 0.01), apple juice (*r* = 0.98, *p* < 0.01), and cran-apple juice (*r* = 0.98, *p* < 0.01), as visually depicted in Figure 2. Starting at 5.4 g of thickener, significant differences were noted between the juice (both samples) and water, with water thickening to a higher degree at a faster pace than either of the juice samples did. Significant differences, although much smaller, were also noted between the apple and cran-apple juice at 6.0 and 6.2 g, and again at 6.8 and 7.0 g.

## 4. Discussion

The results highlight the finding that seemingly small, but carefully controlled alterations in the amount of thickening agent, can impact the physical measures of viscosity in a fairly systematic manner. In this study, the viscosity measurements went from 123.6 cP (thickened apple juice) to 785 cP (modified water), consistent with commonly recommended modifications that range from a nectar (mildly thick) to honey-like (moderately thick) consistency [7,12]. Given that many preparers are inattentive to the product label information [2], the clinical implication is that too much or too little thickening agent (e.g., level vs. heaping teaspoon) could result in a modification that is unsafe for swallowing.

A generally similar pattern of change occurred across different beverages (Figure 2), although the juice products had less thickening with higher levels of starch-based thickener. This may reflect the interaction of the setting time (2 min) and absorption characteristics of a starch-thickening agent when beverages contain other components such as sugar, acid or the small amounts of pectin remaining in bottled juice. Both sugar and acid can reduce viscosity when combined with starch [14]. In this case, the sugar content of the juice may have reduced the water uptake by the starch because both sugar and starch compete for the same water. Acid can result in the breakdown of the starch molecules by the hydrolysis of the glycosidic bonds between the glucose molecules that make up the starch [15,16], although that typically happens over a much longer period of time than the duration implemented in this experiment. The small amount of pectin remaining in clarified bottled juices, such as those used in the experiment, probably would have little impact on thickness.

Small changes of 0.2 g (less than ¼ teaspoon) impacted the physical measurement of viscosity, but it is less clear if those differences are detectable to oral perception [17]. These authors did suggest that at least 4 levels of detectible difference could probably be noted within the nectar-thick category alone. They also noted the exponential relationship of thickener to thickness, which can be seen in this study. That relationship shows that the incremental differences in thickness typically get smaller for each unit of increased thickness. Thus, small incremental differences in the thickener are more important at lower levels than at initially higher levels of thickness. The current study shows that the relationship is beverage dependent. At higher levels of initial thickness, water showed more of an effect from small increases on the thickener than either of the juices did. Chambers et al. [18] showed that the textural properties of beverages of different thicknesses were clearly noted as thicker by descriptive sensory panelists.

## 5. Limitations

We acknowledge that a number of factors may impact the reported findings. One consideration is that we examined one thickening product in dysphagia management, which may not be representative of the thickening patterns of other starch or gum-based thickening agents. However, prior research has shown that over time starch-based thickeners function similarly in many products [19]. Gum-based thickeners have been found to vary from a rapid change (guar gum) to a less rapid change (xanthan gum) in viscosity as the concentration increased [12].

In addition, these samples were tested at room temperature after two minutes of setting time. It is well established that the interaction of a number of factors (base liquid, serving temperature, thickening agent, and thickening time) impacts the viscosity of modified beverages [20,21,22,23]. However, the setting time always resulted in an increase in thickness over time [19], suggesting that these products would also increase in thickness if the time was increased, as might be the case in care settings where products sit before they are fully consumed by patients.

## 6. Conclusions

Our results emphasize the need to properly prepare thickened beverages. A lack of information, support, and belief systems contribute to caregivers’ misuse of thickeners [24,25]. The results from this study further highlight the importance of instruction about modified beverages, especially since error patterns in preparation tend to be repeated across different levels of consistency [2]. For example, care providers who over-thicken nectar (mildly thick) beverages tend to over-thicken honey-like (moderately thick) preparations as well. A common myth held by some care providers, including nutrition specialists, is that “thicker is better” [3]. The practical consequence is that preparers who have this belief may tend to further adjust the amount of thickening agent due to this perception. This data also suggests that, in order to properly thicken a variety of beverages to a consistent level, more information is needed about their standard preparation. In addition to standard education and training practices, simple measurement tools, such as the IDDSI (International Dysphagia Diet Standardization Initiative) flow test [26] or a line spread apparatus offer objective information to practitioners about the thickness of thickened beverages [4,25,27]. Although a more sophisticated research tool was used for this project, simple tools that are reliable and easy to implement have the potential to aid instruction about a target level of consistency and to address quality assurance about the accuracy of day-to-day preparations. The impact of incremental fluctuations in the amount of thickening agent furthers our understanding about the importance of developing more consistent and effective training practices and instituting the use of simple measurement tools in clinical practice for best practices in nutrition care.

## Figures and Tables

**Table 1:** Viscosity measurements in cP for the beverages mixed with incremental increases in the amount of instant thickening agent.

Thickening Agent (in Grams)	Apple Juice Mean (Stf Dev.)	Cran-Apple Juice Mean (Std. Dev.)	Water Mean (Std. Dev.)
5.0 g	123.6 (2.7)	136.9 (8.0)	130.0 (5.4)
5.2 g	151.7 (1.7)	155.8 (9.3)	162.0 (6.2)
5.4 g	168.9 (11.5)	180.5 (5.0)	198.6 (11.3)
5.6 g	199.7 (10.5)	214.7 (7.5)	253.3 (8.8)
5.8 g	221.7 (8.7)	233.9 (7.2)	292.8 (4.1)
6.0 g	265.6 (17.8)	292.8 (9.5)	330.9 (7.2)
6.2 g	293.1 (9.3)	318.3 (21.0)	423.1 (24.2)
6.4 g	358.9 (11.5)	373.1 (15.8)	514.7 (30.1)
6.6 g	397.5 (8.8)	407.2 (14.5)	619.4 (17.9)
6.8 g	445.8 (9.0)	492.8 (26.7)	715.0 (38.8)
7.0 g	492.8 (9.3)	542.2 (5.4)	785.0 (18.6)

## References

[1] Garcia J.M., Chambers E., Molander M. (2005). Thickened Liquids. Am. J. Speech Lang Pathol..

[2] Garcia J.M., Iv E.C., Helverson J., Matta Z., Clark M. (2010). Quality of care issues for dysphagia: Modifications involving oral fluids. J. Clin. Nurs..

[3] Garcia J.M., Chambers E. (2012). Perspectives of Registered Dietitians About Thickened Beverages in Nutrition Management of Dysphagia. Top. Clin. Nutr..

[4] Lam P., Hanson B., Chen J., Duivestein J., Kayashita J., Lecko C., Murray J., Pillay M., Riquelme L., Stanschus S. (2016). Development of International Terminology and Definitions for Texture-Modified Foods and Thickened Fluids Used in Dysphagia Management: The IDDSI Framework. Dysphagia.

[5] Glassburn D.L., Deem J.F. (1998). Thickener Viscosity in Dysphagia Management: Variability among Speech-Language Pathologists. Dysphagia.

[6] Cichero J.A. (2013). Thickening agents used for dysphagia management: Effect on bioavailability of water, medication and feelings of satiety. Nutr. J..

[7] Judson B.L., Sliwinski E., Madson L., Leder S.B. (2012). Promoting Safe Swallowing When Puree is Swallowed Without Aspiration but Thin Liquid is Aspirated: Nectar is Enough. Dysphagia.

[8] Steele C.M., Alsanei W.A., Ayanikalath S., Barbon C.E.A., Chen J., Cichero J.A.Y., Coutts K., Dantas R.O., Duivestein J., Giosa L. (2015). Erratum to: The Influence of Food Texture and Liquid Consistency Modification on Swallowing Physiology and Function: A Systematic Review. Dysphagia.

[9] Robbins J., Gensler G., Hind J., Logemann J.A., Lindblad A.S., Brandt D., Baum H., Lilienfeld D., Kosek S., Lundy D. (2008). Comparison of 2 Interventions for Liquid Aspiration on Pneumonia Incidence. Ann. Intern. Med..

[10] Lotong V., Chun S.S., Chambers E., Garcia J.M. (2003). Texture and Flavor Characteristics of beverages containing commercial thickening agents for dysphagia diets. J. Food Sci..

[11] Matta Z., Chambers E., Garcia J.M., Helverson J. (2006). Sensory characteristics of five beverages prepared with commercial thickeners used for dysphagia diets. J. Am. Dietet. Assoc..

[12] Park J.H., Kim H.-G., Oh B.-M., Lee M.-W., Hwang I.-K., Lee S.-U., Han T.R. (2014). Comparison of Different Gum-Based Thickeners Using a Viscometer and Line Spread Test: A Preliminary Study. Ann. Rehabil. Med..

[13] National Dysphagia Diet Task Force (2008). National Dysphagia Diet: Standardization for Optimal Care.

[14] Gregerson J. (2002). Thick and thin: Tools to manage viscosity. Starches and gums can help to get it just right. Food Process..

[15] Hirashima M., Takahashi R., Nishinary K. (2005). Effects of adding acids before and after gelatinization on the viscoelasticity of corn starch pastes. Food Hydrocol..

[16] Majzoobi M., Beparva P., Farahnaky A., Badii F. (2013). Effects of malic acid and citric acid on the functional properties of native and cross-linked wheat starches. Starch—Stärke.

[17] Steele C.M., James D.F., Hori S., Polacco R.C., Yee C. (2014). Oral Perceptual Discrimination of Viscosity Differences for Non-Newtonian Liquids in the Nectar- and Honey-Thick Ranges. Dysphagia.

[18] Chambers E., Jenkins A., Garcia J.M. (2017). Sensory texture analysis of thickened liquids during ingestion. J. Texture Stud..

[19] Garcia J.M., Chambers E., Matta Z., Clark M., Iv E.C. (2005). Viscosity Measurements of Nectar- and Honey-thick Liquids: Product, Liquid, and Time Comparisons. Dysphagia.

[20] Adeleye B., Rachal C. (2007). Comparison of the Rheological Properties of Ready-to-Serve and Powdered Instant Food–Thickened Beverages at Different Temperatures for Dysphagic Patients. J. Am. Diet. Assoc..

[21] Barbon C.E.A., Steele C.M. (2018). Characterizing the Flow of Thickened Barium and Non-barium Liquid Recipes Using the IDDSI Flow Test. Dysphagia.

[22] Garcia J.M., Chambers E., Matta Z., Clark M., Iv E.C. (2007). Serving Temperature Viscosity Measurements of Nectar- and Honey-Thick Liquids. Dysphagia.

[23] Payne C., Methven L., Fairfield C., Gosney M., Bell A.E. (2011). Variability of starch-based thickened drinks for patients with dysphagia in the hospital setting. J. Texture Stud..

[24] Pelletier C.A. (2005). Feeding beliefs of certified nurse assistants in the nursing home: A factor influencing practice. J. Gerontol. Nurs..

[25] Smith C.H., Jebson E.M., Hanson B. (2014). Thickened fluids: Investigation of users’ experiences and perceptions. Clin. Nutr..

[26] IDDSI (2016). IDDSI Drink Testing Methods—IDDSI FLow Test. http://iddsi.org/framework/drink-testing-methods/.

[27] Garcia J.M., Chambers E., Cook K. (2018). Visualizing the Consistency of Thickened Liquids with Simple Tools: Implications for Clinical Practice. Am. J. Speech Lang Pathol..

